# Supplementary material for: A flexible empirical Bayes approach to multivariate multiple regression, and its improved accuracy in predicting multi-tissue gene expression from genotypes
Source: PLoS Genet. 2023 Jul 7;19(7):e1010539. doi: 10.1371/journal.pgen.1010539 (PMC10355440; doi:10.1371/journal.pgen.1010539)
Supplement: S1 Fig — Each plot summarizes the accuracy of the test set predictions in the 20 simulations for that scenario. The three methods compared were: (1) mr.mash with only “canonical” prior covariance matrices; (2) mr.mash with only “data-driven” prior covariance matrices; and (3) mr.mash with both types of prior covariance matrices. The thick, black line in each box gives the median RMSE relative to the “data-driven” mr.mash RMSE. Since RMSE is a measure of prediction error, lower values are better. Note that the y-axis range varies among panels. (PDF) [file pgen.1010539.s001.pdf]

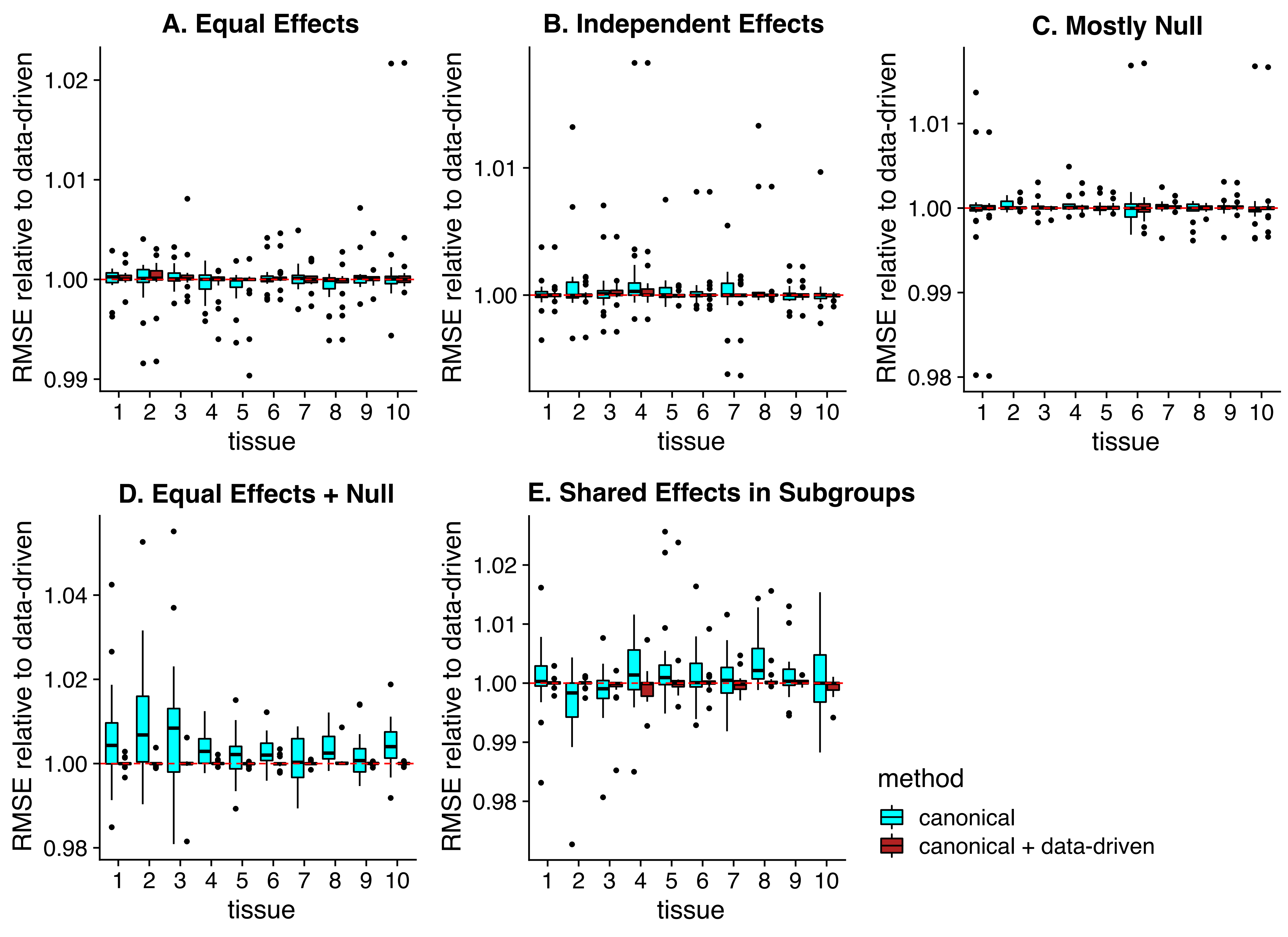

A. Equal Effects
B. Independent Effects
C. Mostly Null
D. Equal Effects + Null
E. Shared Effects in Subgroups
RMSE relative to data-driven
tissue
method
canonical
canonical + data-driven
