# Supplementary figures and images for: A flexible empirical Bayes approach to multivariate multiple regression, and its improved accuracy in predicting multi-tissue gene expression from genotypes
Source: PLoS Genet. 2023 Jul 7;19(7):e1010539. doi: 10.1371/journal.pgen.1010539 (PMC10355440; doi:10.1371/journal.pgen.1010539)

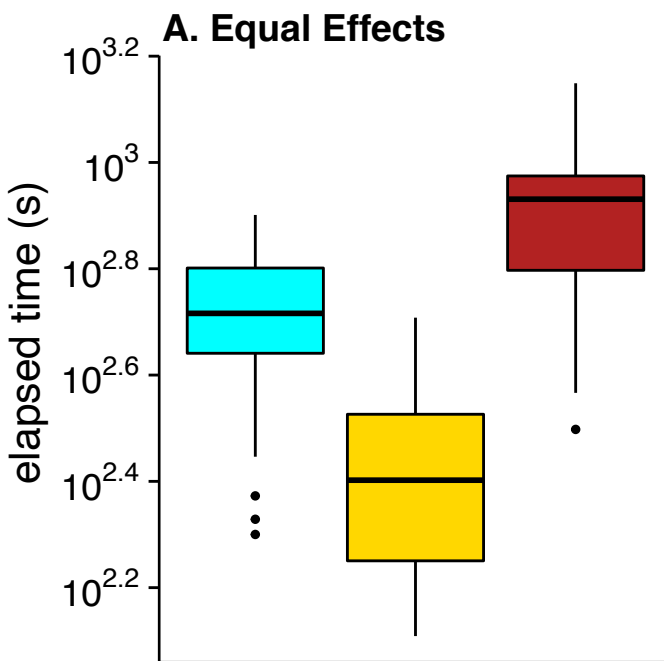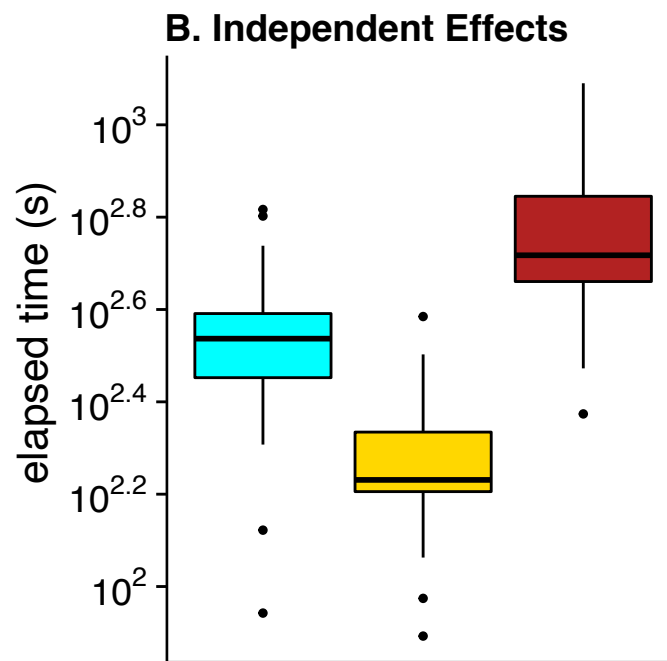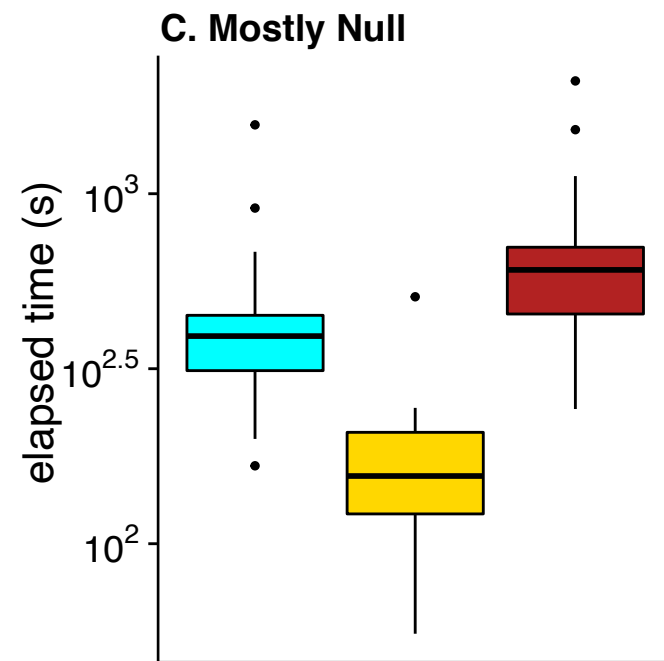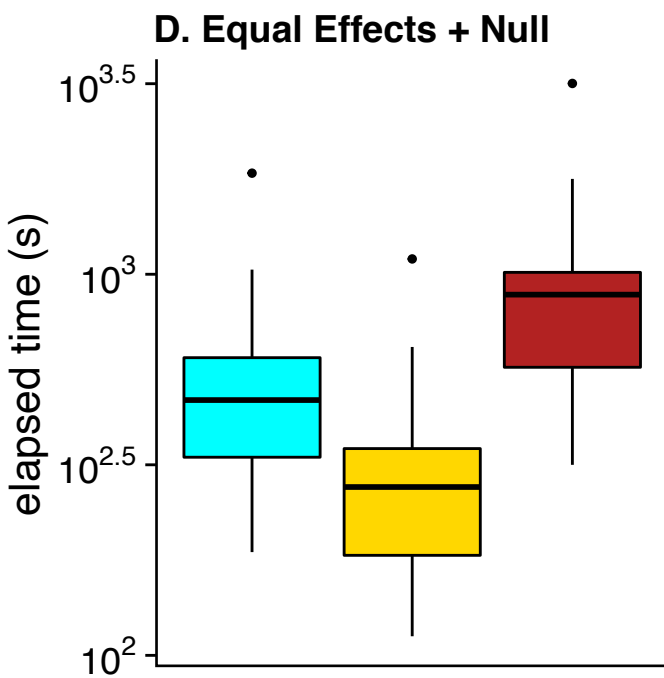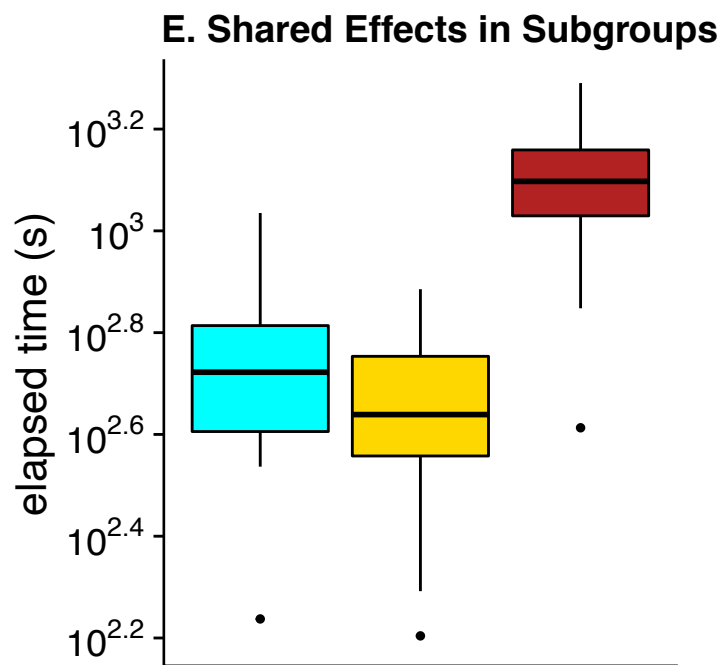

method

- canonical
- data-driven
- both

Supplement: S2 Fig — Each plot summarizes the distribution of model-fitting runtimes for the 20 simulations in that scenario. Note the runtimes did not include the initialization step, which was implemented by running the Group Lasso on the same data set. Once the model fitting was completed, computing the predictions was very fast, so we did not include the prediction step in these runtimes. See S1 Text for the details on the computing environment used to run the simulations. Note that the y-axis range varies among panels. (PDF) [file pgen.1010539.s002.pdf]

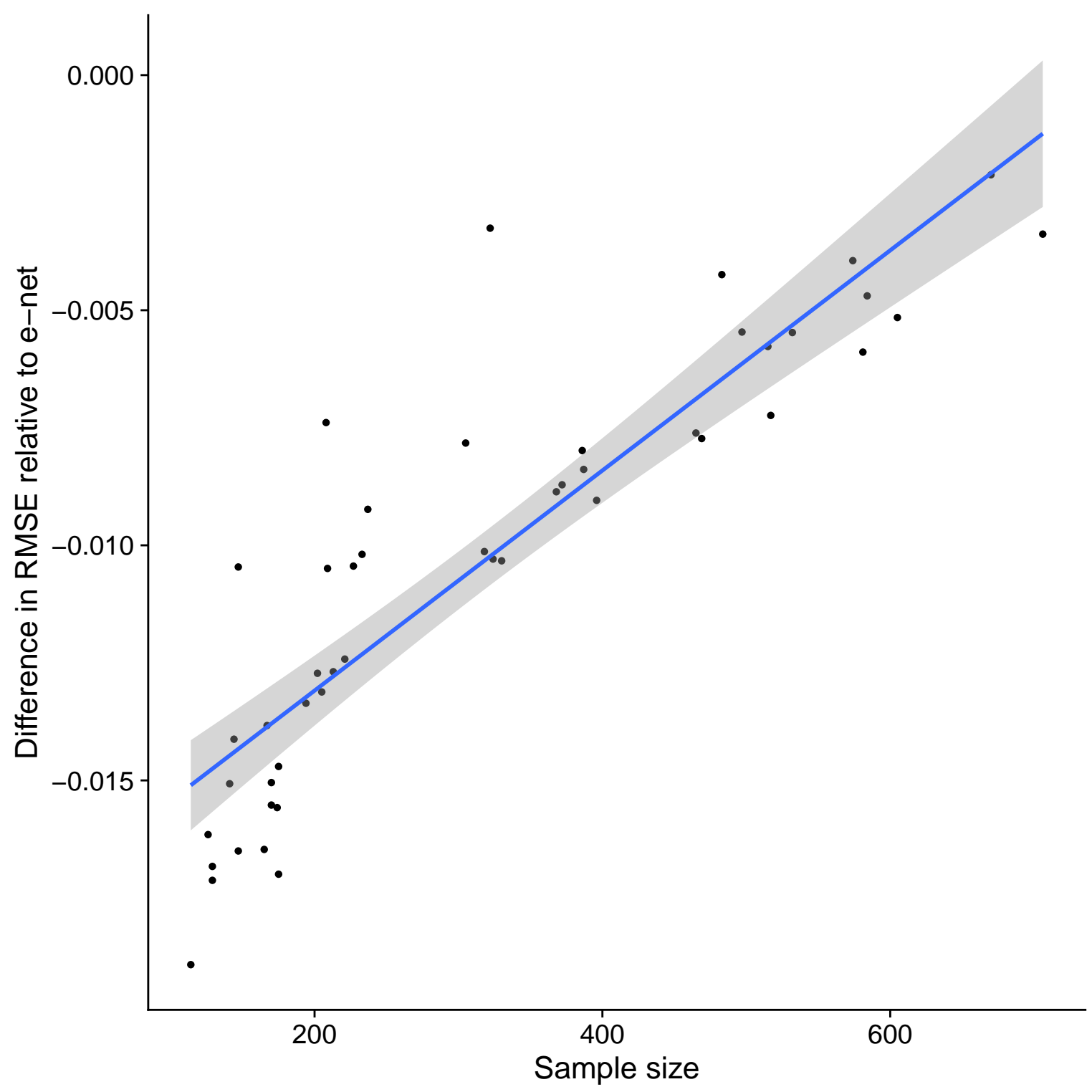

Supplement: S3 Fig — Tissues are plotted along the x-axis by the number of available gene expression measurements and along the y-axis by the improvement in RMSE relative to the Elastic Net; that is, (RMSE(mr.mash) − RMSE(e-net))/RMSE(e-net). (PDF) [file pgen.1010539.s003.pdf]

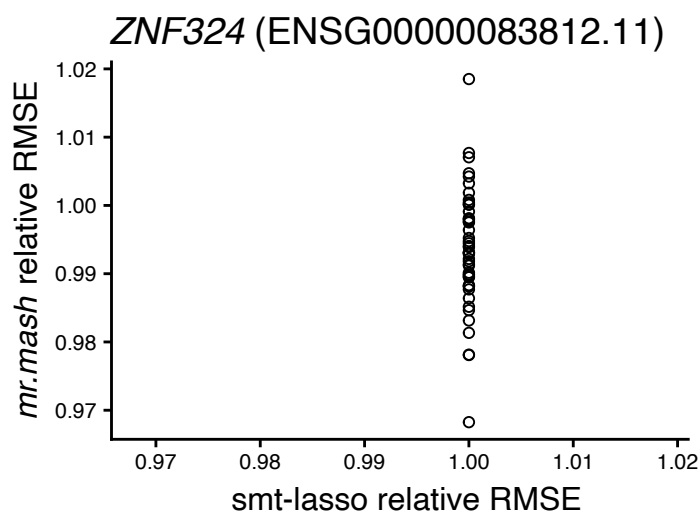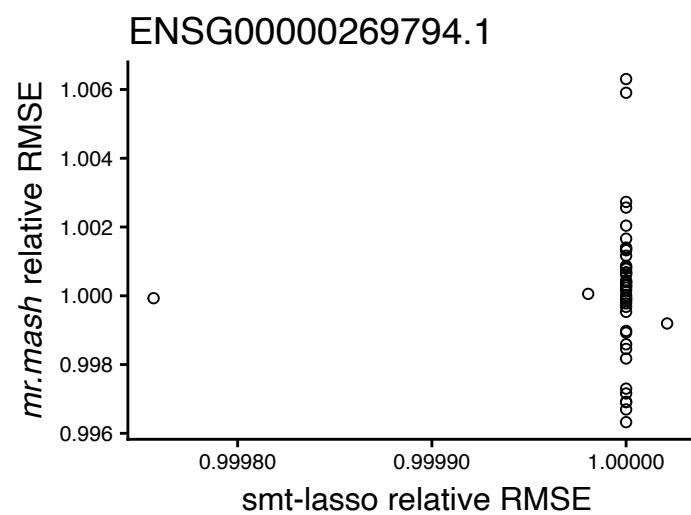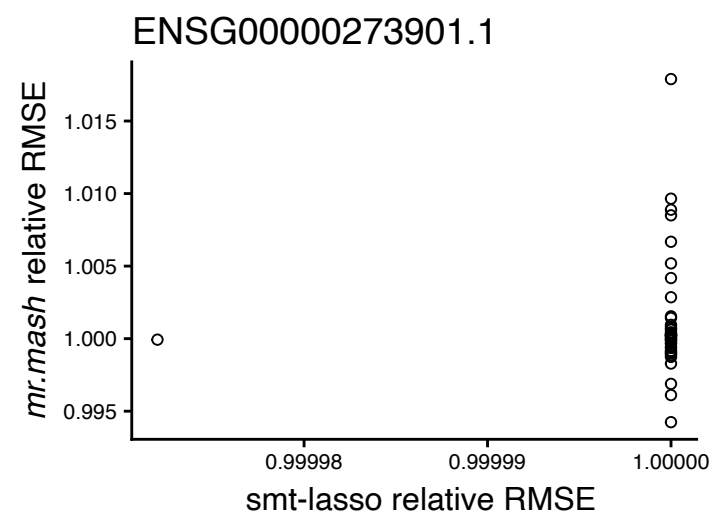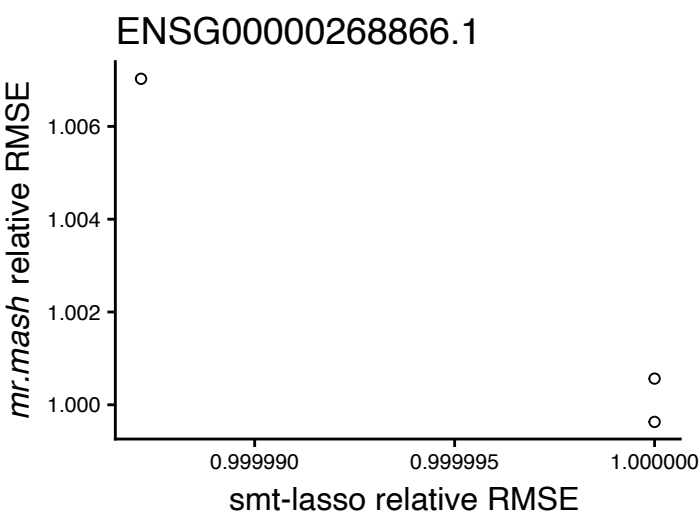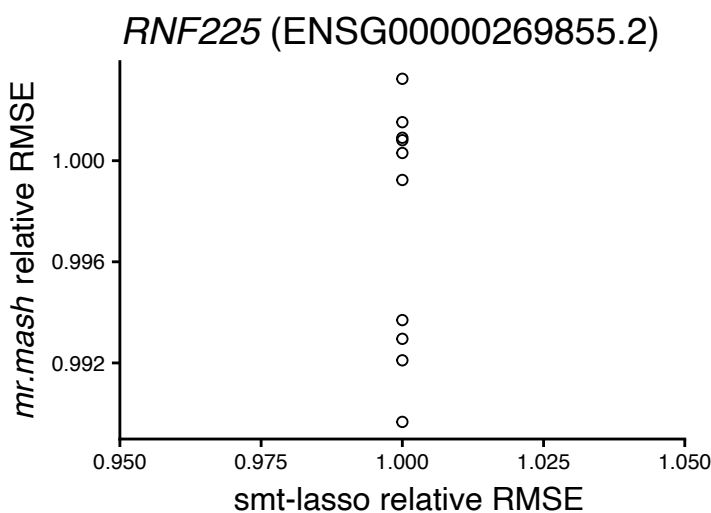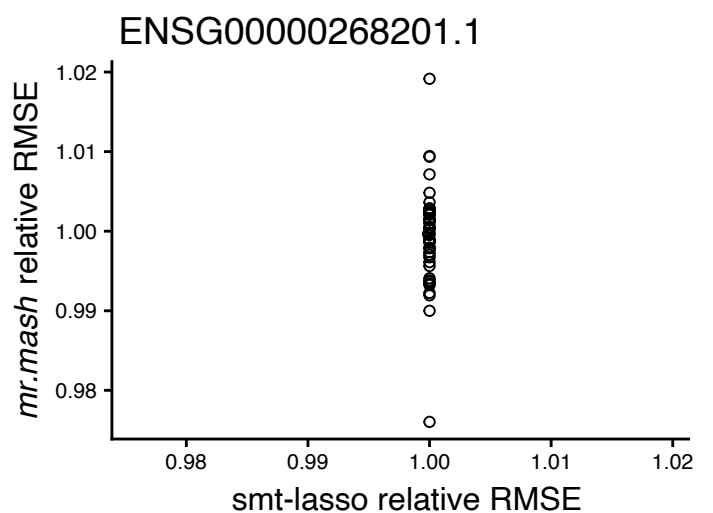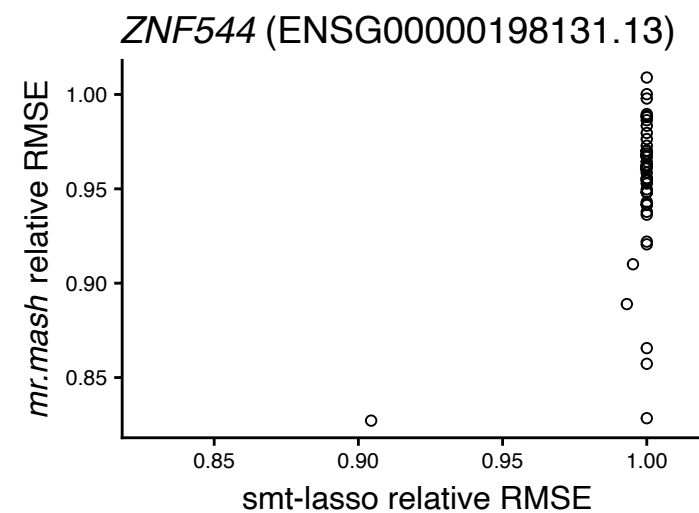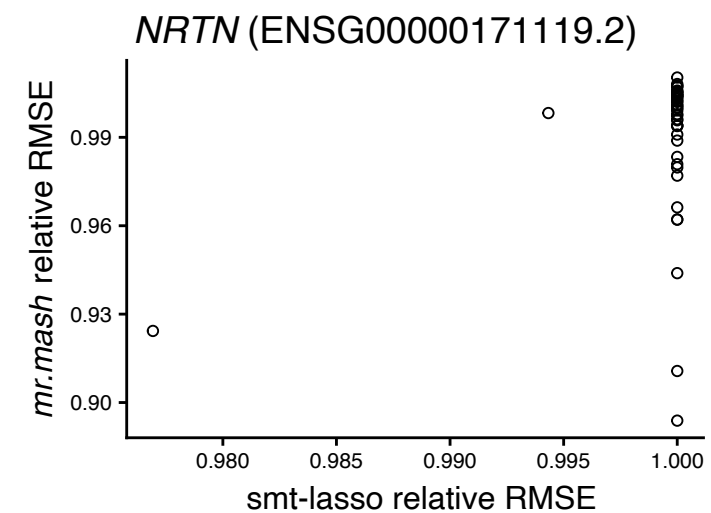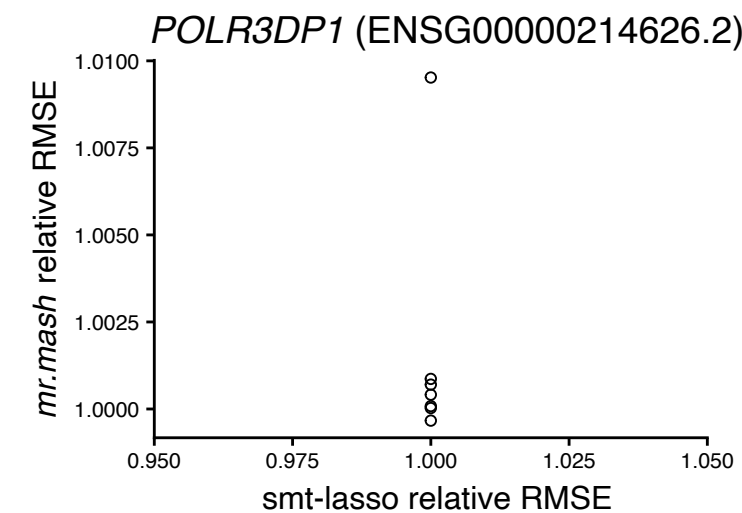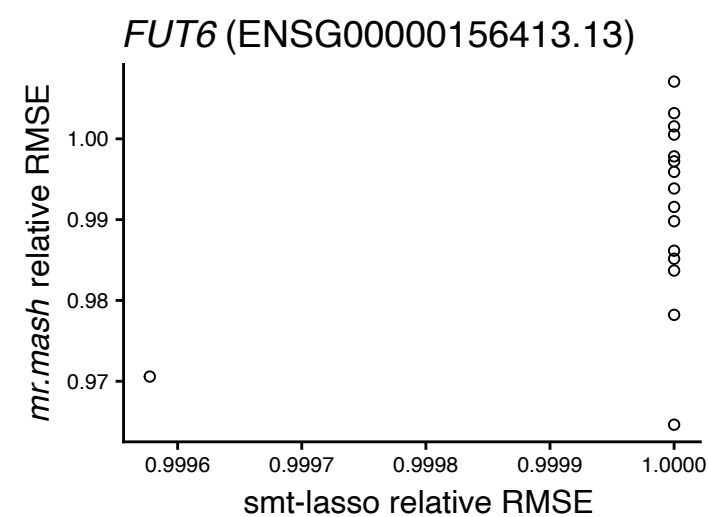

Supplement: S4 Fig — Each plot compares the accuracy of the mr.mash and Sparse Multi-task Lasso gene expression predictions in test samples for a single gene, separately for each tissue. The prediction accuracy is summarized as the RMSE relative to the RMSE that would be obtained by the “naive” predictor in which the genotype has no effect on expression (the naive predictor is therefore simply the mean of the expression measurements in the training data); that is, the x-axis shows RMSE(smt-lasso)/RMSE(naive) and the y-axis shows RMSE(mr.mash)/RMSE(naive). Note that some genes are not expressed in all tissues and so some plots have fewer than 48 points. (PDF) [file pgen.1010539.s004.pdf]
