# Supplementary material for: A flexible empirical Bayes approach to multivariate multiple regression, and its improved accuracy in predicting multi-tissue gene expression from genotypes
Source: PLoS Genet. 2023 Jul 7;19(7):e1010539. doi: 10.1371/journal.pgen.1010539 (PMC10355440; doi:10.1371/journal.pgen.1010539)
Supplement: S5 Fig — Each plot summarizes the accuracy of the test set predictions in 20 simulations for that scenario. Accuracy was quantified by the (standardized) RMSE so that lower RMSE means better accuracy. The two implementations compared are the mtlasso Python software (https://github.com/aksarkar/mtlasso) and the R and C++ implementation used in [59] (this was labeled multi_tissue_twas_sim in the figure because it was downloaded from a git repository with this name, https://github.com/RitchieLab/multi_tissue_twas_sim). Note that the data sets used in this comparison were not the same as the ones used in the main full-data simulations; for this comparison, the data sets were simulated the exact same way except that synthetic genotypes were used instead of the genotypes from the GTEx Project. For more details on this comparison, see [64], in particular the file mrmash_vs_mtlasso_vs_utmost.html. (PDF) [file pgen.1010539.s005.pdf]

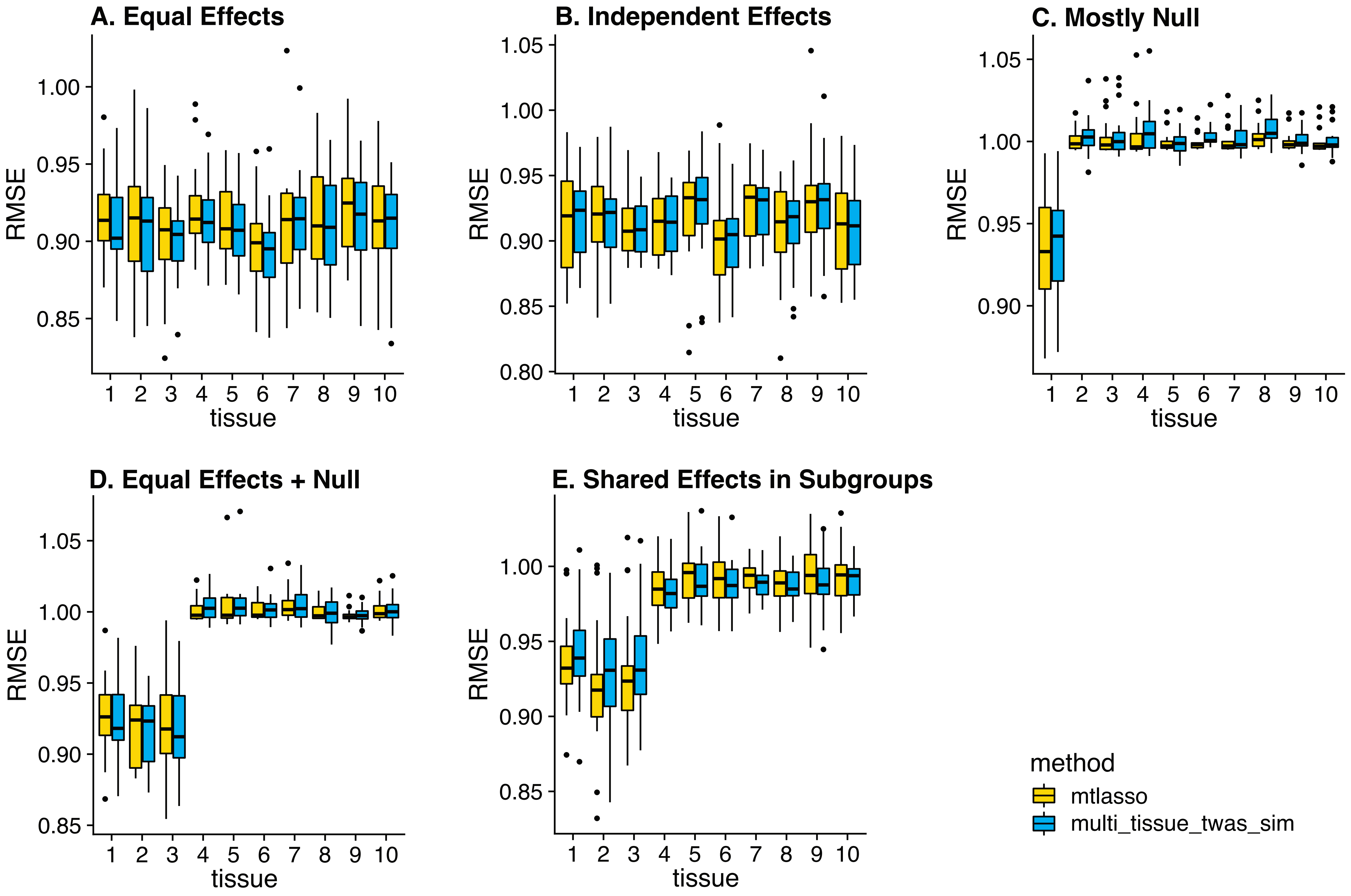
A. Equal Effects
B. Independent Effects
C. Mostly Null
D. Equal Effects + Null
E. Shared Effects in Subgroups
RMSE
tissue
1.05
1.00
0.95
0.90
0.85
0.80
1 2 3 4 5 6 7 8 9 10
method
mtlasso
multi_tissue_twas_sim
