# Supplementary material for: A flexible empirical Bayes approach to multivariate multiple regression, and its improved accuracy in predicting multi-tissue gene expression from genotypes
Source: PLoS Genet. 2023 Jul 7;19(7):e1010539. doi: 10.1371/journal.pgen.1010539 (PMC10355440; doi:10.1371/journal.pgen.1010539)
Supplement: S6 Fig — Each plot summarizes the distribution of model-fitting runtimes for the 20 simulations in that scenario. For details on the methods compared, see the caption for S5 Fig. See also S1 Text for the details on the computing environment used to run the simulations. (PDF) [file pgen.1010539.s006.pdf]

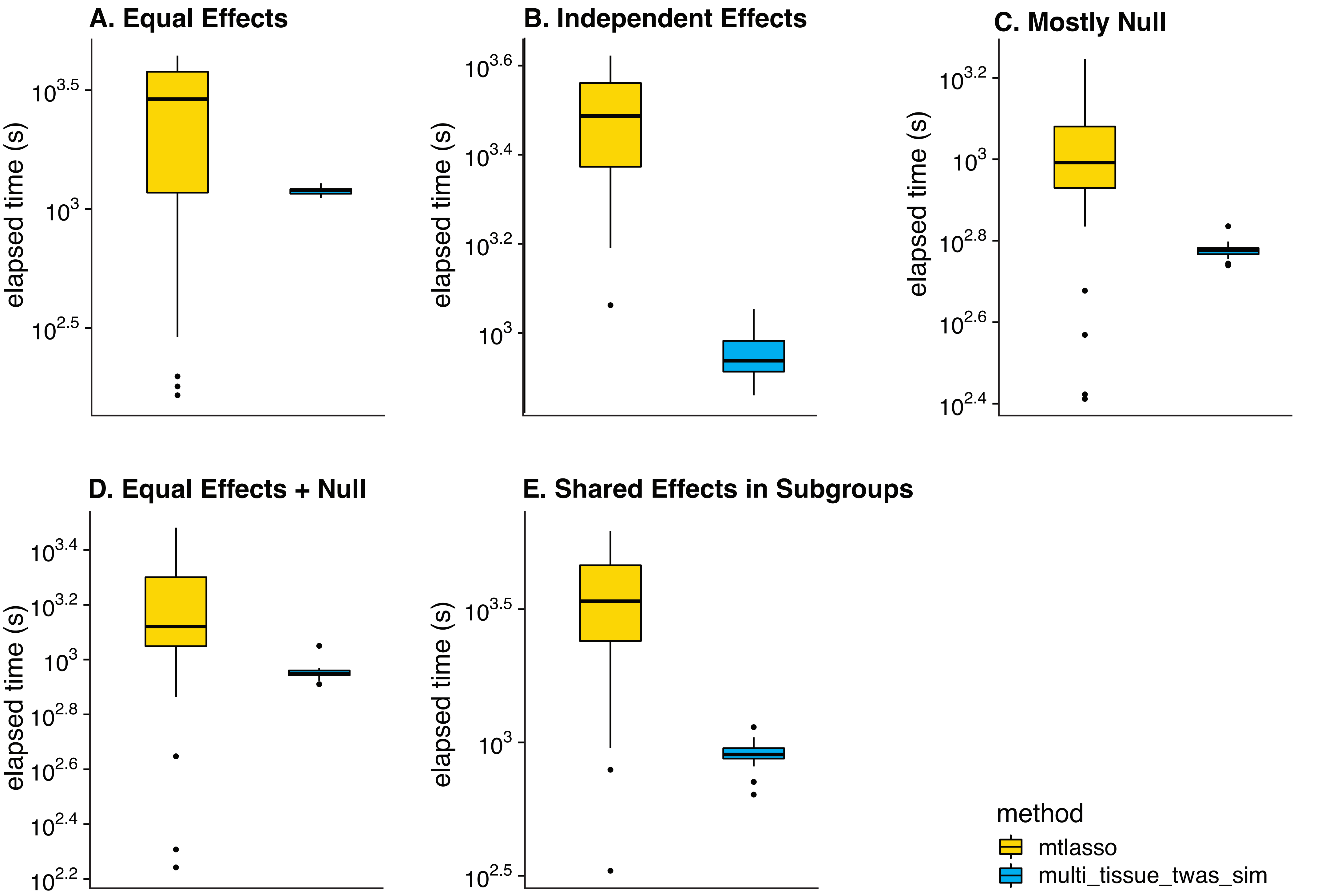

A. Equal Effects
B. Independent Effects
C. Mostly Null
D. Equal Effects + Null
E. Shared Effects in Subgroups
elapsed time (s)
$10^{3.5}$
10^{3}
$10^{2.5}$
$10^{3.6}$
$10^{3.4}$
$10^{3.2}$
$10^{2.8}$
$10^{2.6}$
$10^{2.4}$
$10^{2.2}$
method
mtlasso
multi_tissue_twas_sim
