# Supplementary material for: A flexible empirical Bayes approach to multivariate multiple regression, and its improved accuracy in predicting multi-tissue gene expression from genotypes
Source: PLoS Genet. 2023 Jul 7;19(7):e1010539. doi: 10.1371/journal.pgen.1010539 (PMC10355440; doi:10.1371/journal.pgen.1010539)
Supplement: Attachment — Submitted filename: response_to_reviewers.pdf [file pgen.1010539.s010.pdf]

RESPONSE TO REVIEWS AND SUMMARY OF THE REVISIONS

BY FABIO MORGANTE[1], PETER CARBONETTO[2], GAO WANG[3], YUXIN ZOU[4], ABHISHEK SARKAR[5] AND MATTHEW STEPHENS[6]

[1]*Center for Human Genetics and the Department of Genetics and Biochemistry, Clemson University, Greenwood, SC, USA, fabiom@clemson.edu*

[2]*Department of Human Genetics and the Research Computing Center, University of Chicago, Chicago, IL, USA*

[3]*Department of Neurology and the Gertrude. H. Sergievsky Center, Columbia University, New York, NY, USA*

[4]*Regeneron Genetics Center, Regeneron Pharmaceuticals Inc., Tarrytown, NY, USA*

[5]*Vesalius Therapeutics, Cambridge, MA, USA*

[6]*Departments of Statistics and Human Genetics, University of Chicago, Chicago, IL, USA, mstephens@uchicago.edu*

We thank the reviewers for their thoughtful comments on our manuscript. Here we summarize the main improvements made to the manuscript to address the reviewers' comments:

1. We added more details to "Description of the method," including a new figure (Fig 1) outlining the *mr.mash* algorithm.
2. We ran *mr.mash* on a larger ("whole-genome") data set to demonstrate that it is feasible to apply *mr.mash* to larger data sets, and we reported our results in the Discussion.
3. We compared our missing phenotype imputation strategy with the naive approach of mean imputation.

We have also highlighted in blue the parts of the main text that have changed substantially in the revised version.

Below, comments from the reviewers are written using sans serif font and our responses are in serif.

Comments from Reviewer 1. Morgante *et al* present *mr.mash*, a method for creating genetic prediction models. The key advantage of *mr.mash* is that it is multi-trait, and can share genetic architecture across traits. They first show that *mr.mash* outperforms three rival multi-trait prediction tools on simulated data, then provide comparable results on real data.

Overall, I consider the paper well written, and the proposed model novel. There is also a novel way of handling missing phenotypic values. My main limitation is that *mr.mash* is only demonstrated on small gene expression sized data—600 or so individuals for 10—50 traits, and using what seems to be only a small number of SNPs (I cant find the number mentioned). And therefore, I have no confidence the method can be used on genome-wide data for large numbers of individuals (e.g., 10,000s of individuals, 100,000s SNPs). While I do not consider it a requirement that the data can be applied to large numbers of individuals/SNPs, failure to do so greatly limits the utility of the method. My second limitation, is that the authors do not appear to compare *mr.mash* against state-of-the-art uni-trait methods (I elaborate on this below). On a similar note, my third limitation is that the authors do not appear to compare their new method of imputing phenotypes to the alternative of mean-imputation.

Reviewer 1 major comments.

Major comment 1: I approve of the use of variational bayes (VB), as I found it very effective for my 2021 prediction method (Zhang et al., 2021). In particular, my VB version of

BayesR was many times (50+) more efficient than the MCMC version of BayesR (note that my use of VB was inspired by Bolt-LMM, however, I subsequently saw some of the authors of this paper had championed VB before Bolt-LMM, so I apologise for not being aware of those works earlier). HOWEVER, I experimented with parameter selection based on the approximate likelihood (which I believe is what you do), and found it performed poorly compared to cross-validation. I guess this is also what Bolt-LMM authors found, as they also recommend CV. It may be that for your applications (much smaller), ELBO is fine. However, please can you evaluate the effectiveness of parameter selection by ELBO and CV.

Our response: We thank the reviewer for their detailed comments.

The reviewer suggested that cross-validation (CV) might result in better parameter estimation for *mr.mash*, and therefore suggested that we compare the use of CV against the current approach, which is approximate maximum-likelihood estimation (more precisely, we estimate the parameters by maximizing the ELBO). The issue is that CV is only practical for estimating at most a few parameters. For example, BOLT-LMM, which the reviewer mentions, uses CV to estimate two parameters (this is Step 2a of their algorithm). In particular, they reduce this an to evaluation of 18 combinations of the two parameters (Loh et al., 2015). Similarly for LDAK-BayesR-Predict, CV is performed for 35 combinations of the four parameters $\pi_1, \pi_2, \pi_3, \pi_4$ (Zhang et al., 2021). By contrast, *mr.mash* has many more parameters to estimate: a vector of K mixture weights (this is the vector $\boldsymbol{w}_0$), and an $r \times r$ covariance matrix $\boldsymbol{V}$. In the real GTEx analyses, for example, $r = 48$ and $K > 600$. Therefore, we do not see how CV is feasible for *mr.mash*.

Major comment 2: In the discussion, you suggest *mr.mash* would scale to large sized data, this seems very speculative for me. As discussed above, the number of individuals and SNPs might both be 1,000 times larger, and thus even with linear scaling, 2^9 seconds scaled up is very very large. Further, as you state, the architecture would be very different to gene expression data (*i.e.*, 1–10 causal variants explaining 20% heritability would almost certainly not be realistic), and so this might affect performance/convergence.

Our response: We claimed that *mr.mash* can be applied to "whole-genome" data sets, but did not back up this assertion empirically. To show this, we ran *mr.mash* on a simulated data set with 10 phenotypes, 4,901 individuals and 441,627 SNPs, and we found that the model fitting was able to converge in about 24–50 hours. (These results are included in the Discussion.) Clearly, to apply *mr.mash* to much larger data sets (e.g., UK Biobank), different computational strategies will be needed, such as adapting *mr.mash* to work with "summary data." We comment on this in the Discussion.

Major Comment 3: I believe you should compare *mr.mash* to a leading uni-trait method (instead of only to three other multi-trait methods). As well as being good practice, I note you claim a use of *mr.mash* is for improving the accuracy/power of TWAS, and so a uni-trait analysis would indicate whether this is likely to be the case (because currently, TWAS are based on results from uni-trait prediction models). In terms of which uni-trait method to compare with, personally, I found that LDAK-Bolt-Predict was the best performing uni-trait method, outperforming rival uni-trait methods such as BayesR, Bolt-LMM, BLUP, Lasso, SBLUP, SBAYESR, LDpred, Lassosum, AnnoPred, LDpred-funct and PRS-CS. While you should always be wary of authors finding their own method performs best, I note that Dr. Oliver Pain independently found the same (`https://opain.github.io/GenoPred`). The advantage of LDAK-Bolt-Predict came from incorporating alternative heritability models (in particular, most methods assume the "GCTA Model", whereas I used the "BLD-LDAK Model"). By contrast, it seems *mr.mash* assumes the "van Raden/$\alpha = 0$" model (because you centre, but do not standardize SNPs), which I have found better than GCTA model but inferior to LDAK-Thin or BLD-LDAK model. My guess is that the choice of heritability models has limited impact for your analyses, because you consider very few SNPs. Further, the *mr.mash* model appears to have a good

prior distribution choice. For these reasons, it seems likely *mr.mash* will outperform a leading uni-trait method, but it remains good to confirm.

Our response: The reviewer suggested that we compare *mr.mash* to a leading univariate method, not only leading multivariate methods. The reviewer may have missed the fact that we compared *mr.mash* to a univariate method, *Elastic Net*, in both the simulations and in the real data analysis. However, we do acknowledge that more recent methods such as LDAK-Bolt-Predict (Zhang et al., 2021) may yield better predictions than the *Elastic Net* in some cases. Indeed, there has been much progress in this area in the past few years. We do not claim that the *Elastic Net* is the best univariate regression model; we chose the *Elastic Net* because it is used by PrediXcan, which is a very widely used method for gene expression prediction (Gamazon et al., 2015). We added some text in "Verification and comparison" for clarification:

> The *Elastic Net* was used in the original PrediXcan method for gene expression prediction in TWAS Gamazon et al. (2015), and therefore we view this approach as a baseline univariate regression method for comparison with the multivariate methods. (It is possible that more recent univariate regression approaches with more flexible priors could yield better predictions in this setting, e.g., Zabad, Gravel and Li 2022; Zhang et al. 2021.)

Major comment 4: Please can you compare your clever phenotype imputation method to the naive approach of mean-imputation.

Our response: Following the reviewer's suggestion, in our simulations we included a "naive" imputation approach in which the missing values were set to the mean, then *mr.mash* was run on the imputed $\boldsymbol{Y}$. This method is labeled "*mr.mash* + mean imputation" in Figures 4 and 5. As expected, using the *mr.mash* model to impute missing values was most beneficial in situations where the effects were larger or shared more consistently across tissues, which were also the situations where *mr.mash* was most helpful for improving accuracy.

Reviewer 1 minor comments.

Minor comment 1: I liked the range of simulations, and agreed with your view they covered a wide range of plausible extremes.

Our response: Thank you.

Minor comment 2. I could not find the number of SNPs mentioned (nor an approximate number). I see in the real data, you restricted to within 1 Mb of each gene. This suggests you are analysing about 5-Mb regions, which with sequenced (common?) variants, means about 15,000 variants in each 5-Mb region (but of course, I should not have to guess!).

Our response: The reviewer is correct that we buried information about the number of SNPs in the S1 Text. We have now added this detail to the description of the simulations ("Simulations using GTEx genotypes").

Minor comment 3: Runtime: I find reporting time as log-base 2 seconds quite hard to interpret! Maybe minutes would be more clear?

Our response: As suggested, we revised the runtime plots (Figures 3 and 5) so that seconds are shown on the log-10 scale.

Minor comment 4: Figures: I appreciate you mention y-axes can vary, this is very upfront of you. However, given they do not vary much (IMO), would it be better to fix them to the same values?

Our response: We agree with the reviewer that it is often better to use the same y-axis limits for comparing results across plots. However, in this case it is more important to compare different methods within the same plot (that is, the same simulation scenario), and for this reason we used different y-axis limits in each plot so that the reader can see more clearly the differences among the methods.

Minor comment 5: I am unsure what the order of tissues is in Fig 5, but it seems median RMSE difference. Would it make more sense to instead rank by number of individuals (I personally prefer agnostic ordering, rather than ordering based on results).

Our response: In Fig 6 (previously, Fig 5), we ordered the tissues by median improvement in RMSE—with greatest improvements shown on the left-hand side of the plot—in part to highlight the trend that tissues with smaller sample sizes and more sharing of effects (notably brain tissues) typically showed the greatest improvements.

Minor comment 6: It seems a bit weird to focus on the whole-sequencing data in GTEx—gives the impression you want to restrict the number of individuals, or want very dense data.

Our response: Regarding the use of genotypes from whole-genome sequencing, this is the only available genotype data from GTEx release v8.

Minor comment 7: On p. 6, if you report standardized RMSE, why not just report correlation? Those two might be equivalent (but latter is perhaps easier to explain and more intuitive).

Our response: Regarding the use of correlation instead of RMSE to measure prediction error, the problem with correlation is that it will ignore errors in bias and scale. For example, these two univariate linear regressions will yield the same correlation even though they are very different models: $y = 1 + xb + e$ and $y = 2xb + e$.

Minor comment 8: On p. 6, I am generally satisfied with your choice of comparison methods, except the sentence "For the Elastic Net, the mixing parameter alpha was set to 0.5." I am not familiar with your references, but this seems a strange choice; e.g., when I used `glmnet`, I thought I was advised to use a range of values (e.g., something like 0.5, 0.1 and 0.01), and I am feel this is normally what I see in applications.

Our response: Regarding the choice of α in the Elastic Net penalty term, it is true that α is a term that can also be tuned via CV, similar to λ. The α controls the tradeoff between the Lasso penalty (ℓ_1-norm) and the ridge penalty (ℓ_2-norm). Since we have simulated data sets in which the ground-truth effects are sparse, α mainly serves to avoid degeneracies caused by very strong correlations between SNPs. In fact, Gamazon et al. (2015) found that "LASSO performed similarly to Elastic Net… for subsequent analyses, we focused on the prediction models using Elastic Net because we found it to perform well and to be more robust to slight changes in input SNPs." In PrediXcan, α was set 0.5 (Gamazon et al., 2015). We also now explain this choice in the S1 Text.

Minor comment 9; I appreciated your sensitivity analysis regarding prior covariance matrix.

Our response: Thank you.

Minor comment 10: Some typos: p. 5, "the cuasal variants"; p. 6, "Shared Sffects in Subgroups".

Our response: Thank you for pointing out these typos in the text. They are now fixed in the revised manuscript.

Comments from Reviewer 2. The authors proposed a Bayesian multivariate mixture regression model to jointly predict multiple phenotypes using genotype data (e.g., gene expression in 48 tissues in GTEx). The main contribution is the coordinate descent algorithms via the variational inference and the use of data-driven prior to capture covariance between the target tasks (e.g., sharing effect among GTEx tissues). Overall, the paper is well written and easy to follow. The *mr.mash* method was also described in great amount of details in the supplementary text. The experiments using simulation and GTEx data were also well justified. I have several major comments or suggestions for the authors to consider.

Reviewer 2 comments on the Description of the method.

Comment 1: The description of the method is too brief. The technical contributions of the method were not well highlighted in my opinion. I suggest write down the key EM variational closed-form updates in the method description section of the main text. In particular, in E-step, consider adding closed-form update for the mean and covariance of the effect size $\boldsymbol{b}_j$

(20, 21) but changed it to the actual update (not the simple regression) for $\bar{\boldsymbol{b}}_j$ and $\boldsymbol{S}_j$ and, in M-step, the key update step for $\boldsymbol{V}$ (12) and mixing proportion. The derivation can stay in the supplement but the method should be self-contained in the main text for reproducibility.

Our response: Thank you for your suggestions for clarifying the method description. In the revised manuscript, we have included a sketch of the main algorithm (Fig 1). We have tried to strike the right balance between providing some intuition for the *mr.mash* method without overwhelming with mathematical detail. (Detailed derivations are of course provided in the S1 Text.)

Comment 2: In the main text, it would be also helpful to describe the intuition of the closed-form updates. For example, the mean-field update of the mean effect size of SNP j takes into account the correlation between SNP j and the residual error $\bar{\boldsymbol{R}}_j$ when regressing out the effects of all SNPs except SNP j as well as the covariance between traits.

Our response: Please see our response to Comment 1.

Comment 3: Does *mr.mash* work with summary statistics? I think it can at least when assuming the data are completely observed. That will also improve scalability of the method.

Our response: In principle, *mr.mash* can work with summary statistics, which is potentially useful for data sets with large n. However, this is not currently implemented in the software. We added a note to the Discussion about this. (We are working on a summary-data version of *mr.mash*.)

Reviewer 2 comments on the Verification and comparison.

Comment 1: Although authors briefly discussed this, how does *mr.mash* perform differently on sparse and dense LD in simulation and/or real data?

Our response: We weren't quite sure how to interpret the comment about "sparse and dense LD." Here we assume the reviewer is referring to LD in small genomic regions containing many SNPs in strong LD ("dense LD") versus LD across the genome, in which case most SNP pairs will have weak or no LD between them ("sparse LD"). Since *mr.mash* uses an approximation to the posterior in which the regression coefficients $\boldsymbol{b}_j$ of different SNPs j are independent (see eq. 9 in the S1 Text), this approximation will be most accurate when the SNPs are independent or close to independent in the data (*i.e.*, LD close to zero). Another effect of LD is on *speed of convergence.* The co-ordinate ascent updates over the SNPs j (the for-loop over j in Fig 1) converge most quickly to a fixed point when the SNPs are independent or close to independent; by contrast, these co-ordinate ascent updates can often progress very slowly when SNPs are in strong LD with each other. We do not discuss these aspects in this paper because we have investigated them in detail in some of our previous papers (Carbonetto and Stephens, 2012; Kim et al., 2022).

Comment 2: On line 232, in Independent Effects scenario, authors wrote "multivariate methods do still have some benefits because they can more accurately identify the causal variants." But as we know and as authors mentioned as well, accurate prediction does not imply accurate fine-mapping. Therefore, I don't think this point is a valid in justifying why *mr.mash* performs better in the Independent Effects scenario than the univariate methods.

Our response: The reviewer is correct that *mr.mash* does not identify the causal variants. However, here we are using "causal variant" as a shorthand, as we explained in the text. (We wrote, "We use 'causal variant' as shorthand for 'genetic variant having true non-zero effect in the linear regression for at least one tissue'; that is, $\boldsymbol{b}_j \neq \boldsymbol{0}$.") However, we added a bit of clarification to this statement to avoid possible confusion.

Comment 3: Fig 2: please change seconds to minutes or hours for the ease of reading the runtime.

Our response: Seconds is a standard unit used to report runtimes, however to improve the plots (Figures 3 and 5), we switched from the base-2 log-scale to base-10.

Comment 4: As scalability is a main highlight of the method, what's the computing time as a function of the number of SNPs, number of tasks, and sample size?

Our response: We briefly mentioned in the main text that *mr.mash* scales linearly in n (the number of samples) and p (the number of genetic variants), and either quadratically or cubically in r, the number of tissues/traits (here, the complexity depends on whether $\boldsymbol{X}$ is standardized and whether $\boldsymbol{V}$ is updated or not). In more detail, the computational complexity of a single update to all the parameters (a single outer-loop iteration of the algorithm in Fig 1) is $O(p(nr + kr^3) + nr^2)$ if $\boldsymbol{X}$ is not standardized. If $\boldsymbol{X}$ is standardized, the complexity reduces somewhat to $O(p(nr + kr^2) + nr^2 + kr^3)$. The computational complexity of the missing data imputation step in Algorithm 2 in $O(nr^3)$. We have included these details and other details in the revised S1 Text.

Comment 5: In line 289, authors wrote "*mr.mash* scales either quadratically or cubically in r." When is it quadratic and when is it cubic? I thought that it is always cubic because of inverting the covariance matrix $\boldsymbol{V}$.

Our response: Please see our response to Comment 4.

Comment 6: What's the exact time complexity of *mr.mash*?

Our response: Please see our response to Comment 4.

Reviewer 2 comments on the Applications.

Comment 1: Authors experimented on 1,000 randomly chosen genes. It would be useful to examine three more choices: (1) tissue-specific genes (*i.e.*, similar to the Mostly Null scenario); (2) highly variable genes (*i.e.*, similar to the shared effects in subgroups scenario); (3) most heritable genes across tissues (*i.e.*, examining the upper bound of prediction performance)

Our response: This was precisely our intention behind the simulations, where we designed five different simulation scenarios in order to evaluate *mr.mash* and the other methods in a range of settings. The fundamental problem is that it is not clear how to define these categories for the real data since we don't know the truth. We could of course run *mr.mash* to estimate these categories, but that would be circular reasoning.

Comment 2: Line 354, authors wrote "the improvements tended to be greatest for tissues with more sharing effects and for tissues with smaller sample sizes (Fig 5, S3 Fig)." Fig 5 only supports the latter claim on the sample size but not the form claim on the sharing effects. Similar to S3 Fig, I suggest plot delta RMSE as a function of average covariance computed from Fig 6 to see whether there is a linear trend.

Our response: We agree that this statement was too strong, and the support for such a claim is only anecdotal. For example, *mr.mash* yielded the greatest performance gain for *substantia nigra* because the sample size for that tissue was small and it had high effect sharing with other brain tissues. We revised the text accordingly.

Comment 3: For Fig 6, please label *substantia nigra* to help the reader find it when being referred to this figure in the main text.

Our response: Thank you for the suggestion to highlight *substantia nigra* in Fig 6 (now Fig 7). We have now highlighted this tissue and other tissues mentioned in the text in Figures 6 and 7 to better draw attention to the results in the figure.

Comment 4: Line 372: authors commented on the tendency of smt-lasso to overshrink effect size. By looking at S4 Fig, smt-lasso is not much different from taking the mean of the gene expression, implying that only the unpenalized bias term in the linear model was fit and all regression coefficients are essentially zeros. Would this be caused by large penalty lambda weights, which are supposed to be selected via a validation set?

Our response: One possible explanation for the worse than expected performance of the *Sparse Multi-task Lasso* was the use of a simple approximation suggested in the UTMOST

paper (Hu et al., 2019) to address the issue that the updates are very time consuming when some entries of $\boldsymbol{Y}$ are missing. We speculate that this approximation may be poor in settings where the effect sizes are small and many entries of $\boldsymbol{Y}$ are missing. We describe this approximation in Sec. E.3 of the S1 Text.

Comment 5: As authors cited, Hu et al. (2019) described a method called UTMOST,which was applied to GTEx. While authors experimented their own implementation of smt-lasso, it is worthwhile to directly experiment with UTMOST (`https://github.com/Joker-Jerome/UTMOST`).

Our response: In the S1 Text, we now discuss the different implementations of the *Sparse Multi-task Lasso*, highlighting why we did not use the UTMOST implementation—see Sec. E.3, "Sparse Multi-task Lasso". In comparisons on simulated data sets without missing data, our implementation of the *Sparse Multi-task Lasso* achieved similar performance to the *Sparse Multi-task Lasso* implemented for Li et al. (2021) (see S5 Fig, S6 Fig).

Reviewer 2 comments on the Supplementary Text.

Comment 1: Is MAF of 0.05 based on GTEx or 1000 Genomes?

Our response: The minor allele frequencies (MAFs) are the sample MAFs calculated from the GTEx genotypes used in this paper. We added a note in the S1 Text to clarify this point.

Comment 2: Algorithm 1, for lines 10, 15, and 16, please append the equation numbers, which I believe should be eqs. 11, 47 and 12, respectively.

Our response: Thank you. We have added the equation references as suggested.

Comment 3: Also, I felt that using the subscript of "1" in Algorithm 1 is confusing where subscript j should have been used for each variant j.

Our response: Our convention has been to use the "0" subscript to indicate priors and the "1" subscript to indicate posteriors. We have clarified this convention in the S1 Text (see "Conventions used in mathematical expressions"). To clarify the algorithm, we have added the j subscripts.

Comment 4: For eq. 11, please write down the closed-form update equation.

Our response: The closed-form expressions for (11) are given in Sec. F.4, and further details are provided in Proposition 5.

Comment 5: Sec. E.4.3: The authors mentioned that they used `flashr` to generate the data-driven prior covariance and the number of covariance matrices produced differ depending on the simulation setting. For Equal effects and Equal Effects + Null, the pipeline somehow generates 9 matrices. What do those 9 matrices represent?

Our response: The covariance matrices in the mixture prior (eq. 5 in the main text) are intended to capture a variety of effect-sharing patterns. We distinguish two main types of effect sharing patterns, "canonical" and "data-driven", and they are determined in different ways; this is described in detail in Sec. E.4 of the S1 Text.

Comment 6: Related to Point 5, when applied to the GTEx data (when the ground-truth effects are not known), they said each run produced between 33 and 35 data-driven covariance matrices. What are these? Does each covariance correspond to a distinct component so in other words you ended up 33–35 mixture components? I think this is one of the most important contributions and need elaboration.

Our response: Please see our response to Comment 5. We did not emphasize this aspect in the paper because these ideas are borrowed directly from Urbut et al. (2019) with only slight changes. However, as an illustration, in Fig 7 we showed the covariance matrix with with the largest weight in the prior.

Comment 7: What's "Extreme Deconvolution" algorithm?

Our response: Apologies, we did not properly cite the paper describing Extreme Deconvolution (Bovy, Hogg and Roweis, 2011), and it is now cited in the revised S1 Text.

Reviewer 2 minor comments.

Comment 1: Typo in line 171: “Sffects”.

Our response: Thank you for pointing this out. We have corrected this typo in the revised manuscript.

Comment 2: Line 314: “may need improving” $\rightarrow$ “may need improvement”.

Our response: Thank you for pointing this out. We have corrected this typo in the revised manuscript.

REFERENCES

Bovy, J., Hogg, D. W. and Roweis, S. T. (2011). Extreme Deconvolution: inferring complete distribution functions from noisy, heterogeneous and incomplete observations. *Annals of Applied Statistics* **5** 1657–1677.

Carbonetto, P. and Stephens, M. (2012). Scalable variational inference for Bayesian variable selection in regression, and its accuracy in genetic association studies. *Bayesian Analysis* **7** 73–108.

Gamazon, E. R., Wheeler, H. E., Shah, K. P., Mozaffari, S. V., Aquino-Michaels, K., Carroll, R. J., Eyler, A. E., Denny, J. C., Nicolae, D. L., Cox, N. J. and Im, H. K. (2015). A gene-based association method for mapping traits using reference transcriptome data. *Nature Genetics* **47** 1091–1098.

Hu, Y., Li, M., Lu, Q., Weng, H., Wang, J., Zekavat, S. M., Yu, Z., Li, B., Gu, J., Muchnik, S. et al. (2019). A statistical framework for cross-tissue transcriptome-wide association analysis. *Nature Genetics* **51** 568–576.

Kim, Y., Wang, W., Carbonetto, P. and Stephens, M. (2022). A flexible empirical Bayes approach to multiple linear regression and connections with penalized regression. *arXiv* **2208.10910**.

Li, B., Veturi, Y., Verma, A., Bradford, Y., Daar, E. S., Gulick, R. M., Riddler, S. A., Robbins, G. K., Lennox, J. L., Haas, D. W. and Ritchie, M. D. (2021). Tissue specificity-aware TWAS (TSA-TWAS) framework identifies novel associations with metabolic, immunologic, and virologic traits in HIV-positive adults. *PLoS Genetics* **17** e1009464.

Loh, P.-R., Tucker, G., Bulik-Sullivan, B. K., Vilhjálmsson, B. J., Finucane, H. K., Salem, R. M., Chasman, D. I., Ridker, P. M., Neale, B. M., Berger, B., Patterson, N. and Price, A. L. (2015). Efficient Bayesian mixed-model analysis increases association power in large cohorts. *Nature Genetics* **47** 284–290.

Urbut, S. M., Wang, G., Carbonetto, P. and Stephens, M. (2019). Flexible statistical methods for estimating and testing effects in genomic studies with multiple conditions. *Nature Genetics* **51** 187–195.

Zabad, S., Gravel, S. and Li, Y. (2022). Fast and accurate Bayesian polygenic risk modeling with variational inference. *bioRxiv*.

Zhang, Q., Privé, F., Vilhjálmsson, B. and Speed, D. (2021). Improved genetic prediction of complex traits from individual-level data or summary statistics. *Nature Communications* **12** 4192.
