# Supplementary material for: A flexible empirical Bayes approach to multivariate multiple regression, and its improved accuracy in predicting multi-tissue gene expression from genotypes
Source: PLoS Genet. 2023 Jul 7;19(7):e1010539. doi: 10.1371/journal.pgen.1010539 (PMC10355440; doi:10.1371/journal.pgen.1010539)
Supplement: Attachment — Submitted filename: response_to_reviewer2.pdf [file pgen.1010539.s011.pdf]

RESPONSE TO REVIEWS AND SUMMARY OF THE REVISIONS

By Fabio Morgante[1], Peter Carbonetto[2], Gao Wang[3], Yuxin Zou[4], Abhishek Sarkar[5] and Matthew Stephens[6]

[1]*Center for Human Genetics and the Department of Genetics and Biochemistry, Clemson University, Greenwood, SC, USA, fabiom@clemson.edu*

[2]*Department of Human Genetics and the Research Computing Center, University of Chicago, Chicago, IL, USA*

[3]*Department of Neurology and the Gertrude. H. Sergievsky Center, Columbia University, New York, NY, USA*

[4]*Regeneron Genetics Center, Regeneron Pharmaceuticals Inc., Tarrytown, NY, USA*

[5]*Vesalius Therapeutics, Cambridge, MA, USA*

[6]*Departments of Statistics and Human Genetics, University of Chicago, Chicago, IL, USA, mstephens@uchicago.edu*

We thank the second reviewer for their feedback on our revisions. We made several more changes to the manucript to address the reviewer's comments, which we summarize here:

1. We added a new figure (Fig 1) giving an overview of how *mr.mash* is used to predict gene expression from genotypes.
2. We have provided more detailed explanations of the steps involved in constructing the *mr.mash* prior (Section E.4.3 in the S1 Text).
3. We put together a detailed vignette illustrating the basic steps of a mr.mash analysis using the `mr.mash.alpha` R package.

We have also highlighted in blue the parts of the main text that have changed substantially in the revised version.

Main comments from Reviewer 2.

Comment 1. I would not recommend use pseudocode as Fig 1. But I do think that the pseudocode is helpful and can be moved to the Method section. Perhaps some figurative illustration like a graphical abstract would help highlight the input and output of the algorithm.

Our response. Following the reviewer's suggestion, we moved the algorithm (previously, Fig 1) to the S1 Text, and we added a new figure (Fig 1) giving an overview of how *mr.mash* is used to predict gene expression from genotypes.

Comment 2. In my comment on the GTEx application: I asked the following question on real data not simulated data and would like to see some efforts in addressing them experimentally.

> Authors experimented on 1,000 randomly chosen genes. It would be useful to examine three more choices:
> 1. Tissue-specific genes (*i.e.*, similar to the Mostly Null effect scenario); for this, authors can use the same way as used in Finucane et al. (2018) to define tissue-specific genes.
> 2. Highly variable genes (*i.e.*, similar to the shared effects in subgroups scenario);
> 3. Most heritable genes across tissues (*i.e.*, examining the upper bound of prediction performance).

This is not circular if only the training data are used. Alternatively, if all the $\sim$20,000 genes are experimented, then we can examine the estimated effect sizes for each gene and group the genes based on scenarios as outlined in the simulation to gain novel insight on the gene regulatory programs. For example, do the tissue-specific estimated effect sizes indeed capture known tissue-specific genes?

Our response. We would like to remind the reviewer that the focus of our paper is the development of new methods for more accurate prediction of complex phenotypes from genotype data. For example, in the abstract we write, "Predicting phenotypes from genotypes is a fundamental task in quantitative genetics... Simulation results show that these new methods are fast and improve prediction accuracy compared with existing methods in a wide range of settings where effects are shared." In particular, gaining novel insights into gene regulatory programs, while an important goal, is not the focus of our paper. Therefore, we respectfully disagree with the reviewer that such experiments are needed.

Additionally, based on these comments, the reviewer still seems to be confused about the results we have presented in the paper, so we would like to take this opportunity to clarify several points:

1. The "Mostly Null" Scenario represents the situation in which the effects of genotype on gene expression are isolated to a single tissue. However, *this is different from tissue-specific genes.* And, in particular, this is not the same as a "specifically expressed gene" as it is defined in Finucane et al. (2018). Therefore, investigating the question "Do the tissue-specific estimated effect sizes indeed capture known tissue-specific genes?" does not make much sense. We have added a couple sentences to the text to clarify possible confusion about this.

2. It is unclear what the reviewer means by "Highly variable genes"; this could mean that gene expression shows a lot of variation across individual genotypes, or it shows a lot of variation across tissues (or both). In any case, neither interpretation accurately corresponds to the "Shared Effects in Subgroups" scenario. What is true about this scenario is that the *effects* of genotype on gene expression are heterogeneous because the multivariate effects were drawn from a mixture of effect sharing patterns.

3. The most heritable genes are not necessarily the genes that would be easiest to predict, so would not reflect an upper bound on performance. For example, we would expect better predictions for the case when a single SNP has a large effect on expression compared to dozens of SNPs having small effects on expression even if the combined effect of the dozens of SNPs is larger (*i.e.*, higher heritability) than the single SNP. Therefore, for the specific task of predicting expression in unseen (test) genotypes, examining heritability would not be helpful.

Comment 3. Related to Comment 2, how robust is the heatmap in Fig 7? In other words, if you fit your method on another randomly sampled 1,000 genes, would the heatmap change?

Our response. Fig 7 showing the top covariance matrix in the prior was included for illustrative purposes and to aid in explaining the results summarized in Fig 6; for example, this covariance matrix captures the strong sharing of effects among brain tissues, and weaker sharing of testis, whole blood and skelelal muscle, which partly explains the performance improvements summarized in Fig 7. Because the estimation of these covariance matrices is mainly used as a way to guide the estimation of multivariate effects, with the end goal of improving predictions, we did not focus on rigorously evaluating the estimation of the covariance matrices; this question was the focus of earlier work that we build on, including Urbut et al. (2019) and Bovy, Hogg and Roweis (2011). However, to provide a partial answer to the reviewer's question, the top covariance matrix, as well as other matrices with the most weight, should capture the primary effect sharing patterns across the GTEx tissues. Therefore, we expect that for the covariance matrix shown in Fig 7, as well as for other covariance matrices with larger weights, the eigenvectors associated with the largest eigenvalues should be robust (invariant) to random sampling of the genes, whereas the eigenvectors associated with eigenvalues closer to zero would be less stable, and could be quite different when estimated from different random gene subsets.

Other comments from Reviewer 2. I added some clarification to my following comments. Overall, the method description should be self-contained or else leave out the unnecessary details.

Comment 4. I wrote, "E.4.3. The authors mentioned that they used flashr to generate the data-driven prior covariance and the number of covariance matrices produced differ depending on the simulation setting. For Equal Effects and Equal Effects + Null, the pipeline somehow generates 9 matrices. What do those 9 matrices represent?" referring to this sentence, "The number of data-driven matrices produced by this pipeline was different for each simulation, although in all the Equal Effects and Equal Effects + Null simulations this pipeline always produced 9 matrices." The 9 matrices should be described in the text as it adds confusion to why there are 9 not say 5 or 11 matrices as it current reads. Also, the GitHub repository referred by the authors has no minimal documentation explaining this point.

Our response. In the revised submission, we have provided a much more detailed explanation of the steps involved in constructing the *mr.mash* prior; please see the updated text in Section E.4.3 of the S1 Text. In particular, we now explain in more detail (1) why we obtain different numbers of data-driven covariance matrices for different data sets; (2) how the different numbers of data-driven should reflect the complexity of the effect sharing across tissues; and (3) briefly, what the ED algorithm is, and why we use it to estimate the covariance matrices in the prior. Note that most of these steps involved in constructing the prior have been described in Urbut et al. (2019) (aside from minor changes we made for this paper), and therefore these steps do not represent a novel scientific contribution. But to help with understanding the *mr.mash* analyses and making the description of the methods here more accessible and self-contained, we have added more detailed descriptions of these steps.

The reviewer also asked about how we should interpret the covariance matrices: "What do these 9 matrices represent?" And later, in Comment 5, "Does each covariance correspond to a distinct component so in other words you ended up 33–35 mixture components?" In the Description of the method, we give a broad answer to this question: "The idea is that the collection of matrices $\mathscr{S}_0$ should be chosen to include a wide variety of potential effect sharing patterns; the estimated $\boldsymbol{w}_0$ should then assign most weight to sharing patterns that are present in the data, and little or no weight on patterns that are inconsistent with the data." Specifically for the data-driven covariance matrices, these covariances are learned from the data. In Sec. E.4.3 of the S1 Text, we write, "Data-driven matrices are, as the term implies, adapted to the data, in contrast to the canonical matrices which are the same for every data set. The idea is that these data-driven covariance matrices should capture the principal patterns of effect sharing patterns across the r tissues." To be specific, those 9 or 33–35 covariance matrices are are the normalized covariance matrices ($\mathscr{U}_0$ in eq. 16, Sec. E.4.1 of the S1 Text) that were expanded by a grid of scaling factors ($\boldsymbol{\omega}$ in Eq 16, Sec. E.4.1 of the S1 Text) to provide a final list of covariance matrices, each of which corresponds to a single mixture component in the prior.

We emphasize again that the estimation of the covariance matrices has been investigated in detail in prior works, in particular in Urbut et al. (2019) and Bovy, Hogg and Roweis (2011). Nonetheless, in the revised text we have attempted to provide more detailed descriptions in the hopes that it will help the reviewer (and other readers) understand better this aspect of the analysis.

Comment 5. In one of my comments, I wrote, "Related to point 5, when applied to the GTEx data (when the ground-truth effects are not known), they said each run produced between 33 and 35 data-driven covariance matrices. What are those? Does each covariance correspond to a distinct component so in other words you ended up 33–35 mixture components? I think this is one of the most important contributes and need elaboration." Authors responded that

the ideas are borrowed from Urbut et al. (2019). Sorry for being meticulous on this but that does not really answer my question. Please add more explanation.

Our response. Please see our response to Comment 4.

Comment 6. What's "Extreme Deconvolution" algorithm? Authors cited Extreme Deconvolution algorithm by Bovy, Hogg and Roweis (2011). Could you further describe the rationale on why the Extreme Deconvolution is used and briefly its core ideas.

Our response. Please see our response to Comment 4.

Comment 7. The GitHub repo has no minimal documentation on how to run the method. Tutorial and vignette are needed.

Our response. We thank the reviewer for this suggestion. We have put together a detailed vignette illustrating the basic steps of a *mr.mash* analysis. It can be found at `https://stephenslab.github.io/mr.mash.alpha/articles/mr_mash_intro.html`.

Comment 8. Authors mentioned "quadratically" but I think it should be just "cubically" since all of the time complexity functions involve cubic of r.

Our response. The reviewer is correct that the running time of *mr.mash* usually scales cubically with r, the number of tissues. However, there is a special case where the computations can be better reused so that the time complexity is quadratic in r: when $\boldsymbol{X}$ is standardized, $\boldsymbol{V}$ is not estimated, and there are no missing values in $\boldsymbol{Y}$. Section D of the S1 Text gives more details on the computational complexity of *mr.mash*. We have revised our statement in the main text to make this a bit more clear (referring the reader to the S1 Text for details).

REFERENCES

BOVY, J., HOGG, D. W. and ROWEIS, S. T. (2011). Extreme Deconvolution: inferring complete distribution functions from noisy, heterogeneous and incomplete observations. *Annals of Applied Statistics* **5** 1657–1677.

FINUCANE, H. K., RESHEF, Y. A., ANTTILA, V., SLOWIKOWSKI, K., GUSEV, A., BYRNES, A., GAZAL, S., LOH, P.-R., LAREAU, C., SHORESH, N., GENOVESE, G., SAUNDERS, A., MACOSKO, E., POLLACK, S., PERRY, J. R. B., BUENROSTRO, J. D., BERNSTEIN, B. E., RAYCHAUDHURI, S., MCCARROLL, S., NEALE, B. M. and PRICE, A. L. (2018). Heritability enrichment of specifically expressed genes identifies disease-relevant tissues and cell types. *Nature Genetics* **50** 621–629.

URBUT, S. M., WANG, G., CARBONETTO, P. and STEPHENS, M. (2019). Flexible statistical methods for estimating and testing effects in genomic studies with multiple conditions. *Nature Genetics* **51** 187–195.
