# Supplementary material for: A flexible empirical Bayes approach to multivariate multiple regression, and its improved accuracy in predicting multi-tissue gene expression from genotypes
Source: PLoS Genet. 2023 Jul 7;19(7):e1010539. doi: 10.1371/journal.pgen.1010539 (PMC10355440; doi:10.1371/journal.pgen.1010539)
Supplement: Attachment — Submitted filename: PGENETICS-D-22-01332 review.docx [file pgen.1010539.s008.docx]

The authors proposed a Bayesian multivariate mixture regression model to jointly predict multiple phenotypes using genotype data (e.g., gene expression in 48 tissues in GTEx). The main contribution is the coordinate descent algorithms via the variational inference and the use of data-driven prior to capture covariance between the target tasks (e.g., sharing effect among GTEx tissues). Overall, the paper is well written and easy to follow. The mr.mash method was also described in great amount of details in the supplementary text. The experiments using simulation and GTEx data were also well justified. I have several major comments or suggestions for the authors to consider.

Major comments:

Comments on the Supplementary text (S1_text):

1. In S1_text, is MAF of 0.05 based on GTEx or 1000 Genome?
2. Algorithm 1, for line 10, 15, and 16, please append the equation numbers, which I believe should be Eq 11, 47, and 12, respectively.
3. Also, I felt that using the subscript of “1” in Algorithm 1 is confusing where subscript “j” should have been used for each variant j
4. For Eq 11, please write down the closed-form update equation.
5. E.4.3. The authors mentioned that they used flashr to generate the data-driven prior covariance and the number of covariance matrices produced differ depending on the simulation setting. For Equal effects and equal effects + Null, the pipeline somehow generates 9 matrices. What do those 9 matrices represent?
6. Related to point 5, when applied to the GTEx data (when the groundtruth effects are not known), they said each run produced between 33 and 35 data-driven covariance matrices. What are those? Does each covariance correspond to a distinct component so in other words you ended up 33-35 mixture components? I think this is one of the most important contributes and need elaboration.
7. What’s “Extreme Deconvolution” algorithm?

Main text:

Methods:

1. The description of the method is too brief. The technical contributions of the method was not well highlighted in my opinion. I suggest write down the key EM variational closed-form updates in Method description section of the main text. In particular, in E-step, consider adding closed-form update for the mean and covariance of the effect size b_j (Eq 20, 21) but changed it to the actual update (not the simple regression) for b_j_bar and S_bar; in M-step: the key update step for V (Eq 12) and mixing proportion. The derivation can stay in the supplement but the method should be self-contained in the main text for reproducibility.
2. In the main text, it would be also helpful to describe the intuition of the closed-form updates. For example, the mean-field update of the mean effect size of SNP j takes into account the correlation between SNP j and the residual error R_j when regressing out the effects of all SNPs except SNP j as well as the covariance between traits.
3. Does mr.mash work with summary statistics? I think it can at least when assuming the data are completely observed. That will also improve scalability of the method.

Simulation Results:

1. Although authors briefly discussed this, how does mr.mash perform differently on sparse and dense LD in simulation and/or real data?
2. On line 232, in Independent Effects scenario, authors wrote “multivariate methods do still have some benefits because they can more accurately identify the causal variants”. But as we know and as authors mentioned as well, accurate prediction does not imply accurate fine-mapping. Therefore, I don’t think this point is a valid in justifying why mr.mash performs better in the Independent Effects scenario than the univariate methods.
3. Fig. 2. Please change seconds to minutes or hours for the ease of reading the runtime.
4. As scalability is a main highlight of the method, what’s the computing time as a function of the number of SNPs, number of tasks, and sample size?
5. In line 289, authors wrote “mr.mash scales either quadratically or cubically in r”. When is it quadratic and when is it cubic? I thought that it is always cubic because of inverting the covariance matrix V.
6. What’s the exact time complexity of mr.mash?

GTEx application:

1. Authors experimented on 1000 randomly chosen genes. It would be useful to examine three more choices:
   a. (1) tissue-specific genes (i.e., similar to the mostly null effect scenario);
   b. (2) highly variable genes (i.e., similar to the shared effects in subgroups scenario);
   c. (3) most heritable genes across tissues (i.e., examining the upper bound of prediction performance)
2. Line 354, authors wrote “the improvements tended to be greatest for tissues with more sharing effects and for tissues with smaller sample sizes (Fig 5, S3 Fig)”. Fig 5 only supports the latter claim on the sample size but not the form claim on the sharing effects. Similar to S 3, I suggest plot delta RMSE as a function of average covariance computed from Fig 6 to see whether there is a linear trend.
3. For Fig. 6, please label substantia nigra to help the reader find it when being referred to this figure in the main text.
4. Line 372: authors commented on the tendency of smt-LASSO to overshrink effect size. By looking at Fig. S4, smt-lasso is not much different from taking the mean of the gene expression, implying that only the unpenalized bias term in the linear model was fit and all regression coefficients are essentially zeros. Would this be caused by large penalty lambda weights, which are supposed to be selected via a validation set?
5. As authors cited, Hu et al (2019) described a method called UTMOST,which was applied to GTEx. While authors experimented their own implementation of smt-lasso, it is worthwhile to directly experiment UTMOST (<https://github.com/Joker-Jerome/UTMOST>).

Minor:

1. Typo in line 171: “Sffects”
2. Line 314: “may need improving” => “may need improvement”
